# Franck-Condon Factors to High Vibrational Quantum Numbers II: SiO, MgO, SrO, AlO, VO, NO

**DOI:** 10.6028/jres.066A.022

**Published:** 1962-06-01

**Authors:** R. W. Nicholls

## Abstract

Franck-Condon factor arrays have been computed numerically to high vibrational quantum numbers for the band systems of the following diatomic oxides of interest in astrophysics and atmospheric physics

SiO: (A^1^Π–X^1^Σ^+^)
MgO: (B^1^Σ–A^1^Π)
MgO: (B^1^Σ–X^1^Σ)
SrO: (A^1^Σ–X^1^Σ)
AlO: (A^2^Σ^+^–X^2^Σ^+^)
VO: (A^2^Δ–X^2^Δ)
NO*β*: (B^2^Π –X^2^Π)
NO*γ*: (A^2^Σ^+^–X^2^Π)

## 1. Introduction

In the first paper of this series [Nicholls, 1961] which is referred to below as (I), the influence of the Franck-Condon factor *q_v′v″_* = |∫*ψ_v′_ψ_v″_dr*|^2^ in determining the probability of a *v′*↔*v″* molecular transition and in particular the intensity of the radiative *v′*↔*v″* transition was discussed.

A review was given of the methods which have been developed since the original work of Condon to compute arrays of the factors for molecular band systems. In particular a method of direct computation of arrays of Franck-Condon factors for “Morse” molecules by numerical integration using an electronic computer program was described and *q_v′v″_*- arrays for a number of transitions of N_2_ and $N_{2}^{+}$ were reported. The program has also been used to compute *q_v′v″_*-arrays for the O_2_ Schumann-Runge [Nicholls, 1960] and $N_{2}^{+}\left({\;}^{2}\prod_{u}-A^{2}\Pi_{u}\right)$ [Nicholls, 1962a] systems.

In the present paper further results from the use of this program are presented for band systems of a number of diatomic oxides.

## 2. Basic Data

The computations were performed upon the 704 electronic digital computer of the National Bureau of Standards using a program written by Miss I. Stegun and Miss R. Zucker of the computation laboratory. The input data for this program are *ω_e_*, *ω_e_x_e_, r_e_, μ*_A_*υ*_max_ for both states of the transition involved. The notation is standard [Herzberg 1950]. The transitions treated here are:

SiO: (A^1^Π –X^1^Σ^+^)
MgO: (B^1^Σ–A^1^Π)
MgO: (B^1^Σ–X^1^Σ)
SrO: (A^1^Σ–X^1^Σ)
AlO: (A^2^Σ^+^–X^2^Σ^+^)
VO: (A^2^Δ–X^2^Δ)
NO*β*: (B^2^Π–X^2^Π)
NO*γ*: (A^2^Σ^+^–X^2^Π)

some of which (VO, AlO, SrO, MgO) are of importance in the spectra of stellar envelopes [Hynek, 1951] and others (NO) of which are of importance in the spectrum of hot air [Daiber and Williams, 1961].

The basic input data for these transitions is listed in table 1. It was taken from the compilation of Herzberg [1950] or from original papers of analysis of the band systems. The *υ*_max_ entries correspond to the highest spectroscopically identified vibrational level of the electronic state involved.

## 3. Results

The eight Franck-Condon factor arrays appear in tables 2 to 9 inclusive. In these rectangular, double entry tables, data are of course given for many more bands than are commonly observed. Entries which are at a local maximum, relative to their neighbors, have been italicized and the loci of these maximum values (Condon loci or “parabolae”) are indicated by dots between the columns. In these tables, the power of 10 by which the entry is to be multiplied is indicated by the negative number in each entry. [Table t3-jresv66an3p227_a1b] [Table t4-jresv66an3p227_a1b] [Table t5-jresv66an3p227_a1b] [Table t6-jresv66an3p227_a1b] [Table t7-jresv66an3p227_a1b] [Table t8-jresv66an3p227_a1b]

## 4. Discussion

Most of the band systems reported here were studied because of a need to interpret in our laboratories spectra produced by shock excitation of powdered oxides [Nicholls and Parkinson, 1957; Parkinson and Nicholls, 1959]. As has been noted above a number of the systems are also of importance in astrophysics and atmospheric physics.

It was pointed out in (I) that the prime limitation in the use of such tables is the realism of the Morse model for the respective molecular potentials involved. Such tables as 2 to 9 are therefore to some extent interim results to be used until comparable tables derived from wavefunctions from numerical “real” molecular potentials become available [Jarmain, 1959, 1960, 1961; Fallon, Tobias, and Vanderslice, 1961; and references therein].

The profiles of primary and subsidiary Condon loci are indicated. They exhibit the same general patterns as were noted in (I); viz: the smaller Δ*r_e_*, the narrower is the primary parabola and the fewer subsidiary loci, while the larger Δ*r_e_* the wider the primary parabola and the more subsidiary loci. Both extremes, and some intermediate cases, are to be seen in tables 2 to 9. Δ*r_e_* is largest (0.26A) in the case of the NO*β* system for which the primary parabola is wide and its vertex is far removed from the origin. The O_2_ Schumann-Runge system [Nicholls, 1960] is a comparable example. The MgO (B^1^Σ–X^1^Σ) system has a narrow primary locus. The A10(A^2^Σ^+^–X^2^Σ^+^) system (Δ*r_e_*=0.049A) is a somewhat similar, though less extreme case.

Knowledge of the profiles (in the *v′, v″* plane) of these loci has obvious important applications to the definitive identifications of bands. Some preliminary remarks were made in (I) on a study of the geometry of the loci which is elaborated upon more fully elsewhere [Nicholls, 1962b, c].

In addition to the results quoted in (I) and in this paper, the computer program has also been used to evaluate *q*-arrays for the O_2_ Schumann-Runge system [Nicholls, 1960], the $N_{2}^{+}{\;}^{2}\Pi_{u}-A^{2}\Pi_{u}$ system [Nicholls, 1962a] and a number of band systems of importance in the vacuum ultraviolet [Nicholls, 1962c]. It also has applications to the evaluation of Franck-Condon factor arrays for excitation and ionization transitions from ground states. Data for some ionization transitions were given in (I) for $N_{2}^{+}$ and by Wacks and Krauss [1961] for $O_{2}^{+}$. In both of these cases only one or two levels of the lower (ground) state involved were employed as the results were to be used for ionization of cold gases. Some recent data have been calculated and will be published elsewhere for complete $\left(0<v^{\prime \prime }<v_{\max}^{\prime \prime };0<v^{\prime }<v_{\max}^{\prime }\right)$ Franck-Condon factor excitation arrays for a number of transitions between N_2_(X^1^Σ) and other states of N_2_ and $N_{2}^{+}$.

In the case of SiO, where an analytic method has also been used to compute a limited array (for application to shock tube spectra) [McGregor, Jarmain, and Nicholls, 1961] agreement between the two arrays is excellent.

## Figures and Tables

**Table 1:** Basic Data

State	*ω_e_*(cm^−1^)×10^−3^	*ω_e_x_e_*(cm^−1^)	*r_e_*(A)	*µA*	*υ*_max_
					
SiO					
X^1^Σ^+^	1.24203	6.407	1.510	10.18013	10
A^1^Π	0.85151	6.143	1.621	10.18013	10
MgO					
X^1^Σ	.7851	5.18	1.749	9.59888	7
A^1^Π	.6644	3.91	1.864	9. 59888	7
B^1^Σ	.8241	4.76	1.737	9. 59888	7
SrO					
X^1^Σ	.6535	4.0	1.921	13.5302	6
A^1^Σ	.624	2.0	2.022	13.5302	6
AlO					
X^2^Σ^+^	.9782	7.12	1.6176	10.0452	7
A^2^Σ^+^	.870	3.80	1.6667	10.0452	7
VO					
X^2^Δ	1.0127	4.9	1.890	12.1768	10
A^2^Δ	0.8635	5.4	2.033	12.1768	10
NO					
X^2^Π	1.90385	13.97	1.1508	7.46881	18
A^2^Σ^+^	2.3713	14.48	1.0637	7.46881	7
B^2^Π	1.03769	7.603	1.415	7.46881	6

**Table 2:** Franck-Condon factors to hiqh vibrational quantum numbers for the *SiO A^1^Π–X^1^Σ* band system

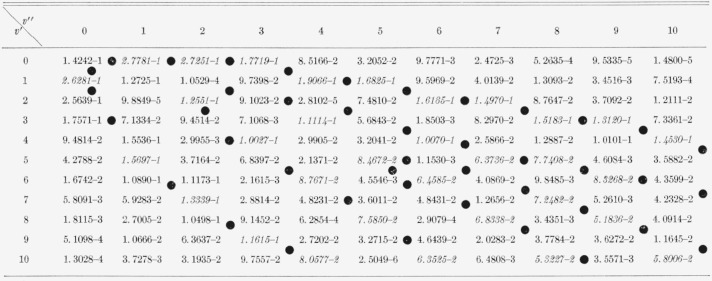

**Table 3 t3-jresv66an3p227_a1b:** Franck-Condon factors to high vibrational quantum numbers for the *MgO A^1^Π–B^1^Σ* band system

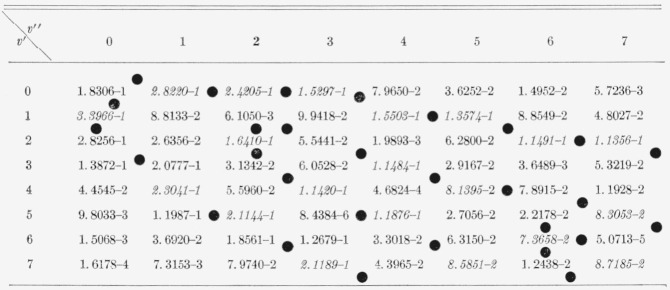

**Table 4 t4-jresv66an3p227_a1b:** Franck–Condon factors to high vibrational quantum numbers for the *MgO B^1^Σ*–band system

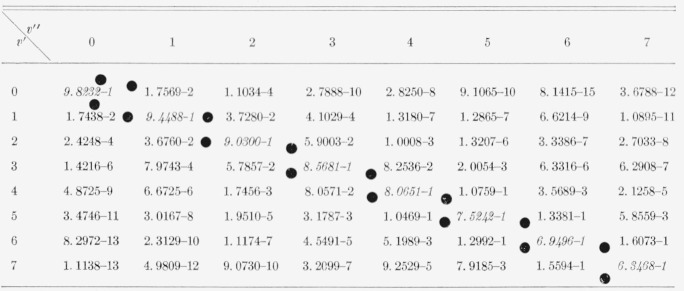

**Table 5 t5-jresv66an3p227_a1b:** Franck-Condon factors to high vibrational quantum numbers for the *SrO A^1^Σ–X^1^Σ* band systems

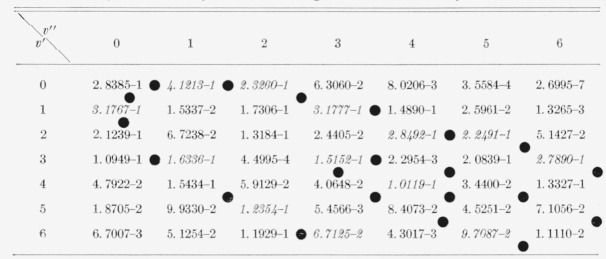

**Table 6 t6-jresv66an3p227_a1b:** Franck-Condon factors to high vibrational quantum numbers for the AlO A^2^Σ–X^2^Σ band system

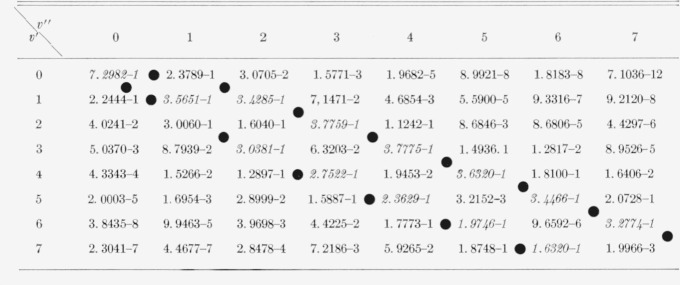

**Table 7 t7-jresv66an3p227_a1b:** Franck-Condon factors to high vibrational quantum numbers for the *VO A^2^Δ–X^2^Δ* band system

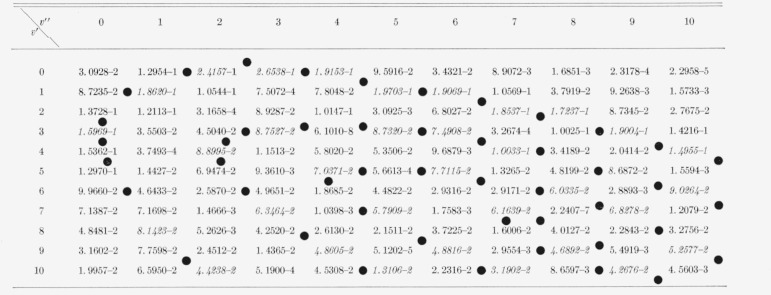

**Table 8 t8-jresv66an3p227_a1b:** Franck-Condon factors to high quantum numbers for the NO beta (B^2^Π –X^2^Π) band system

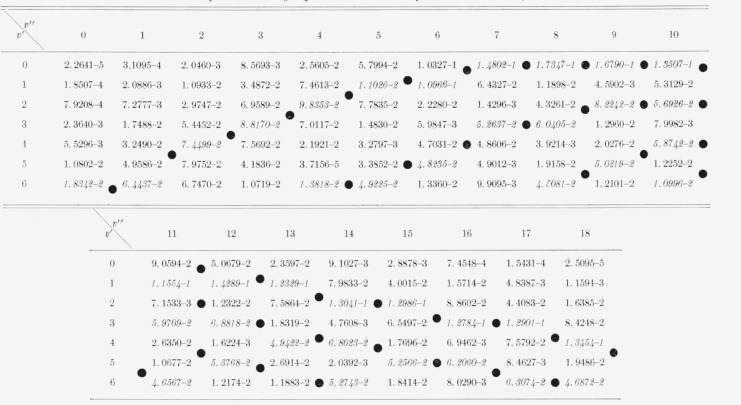

**Table 9:** Franck-Condon factors to high vibration quantum numbers for the no gamma (*A^2^Σ-X^2^Π*) band system

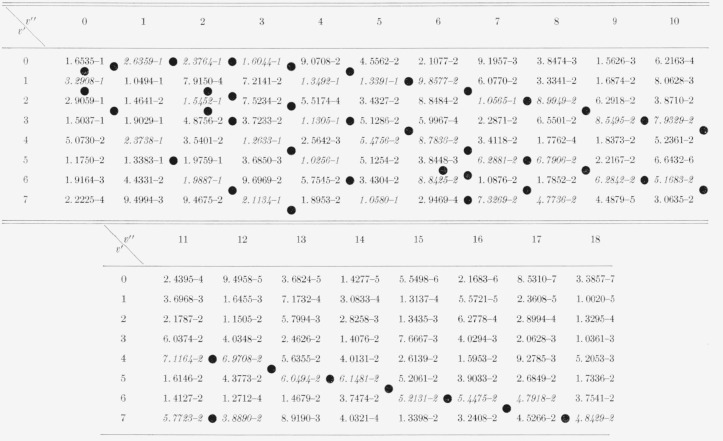
